# Layer-Specific Colocalization of Microglia with Amyloid Plaques in the Middle Temporal Gyrus Predicts Cognitive Decline in Alzheimer's Disease

**DOI:** 10.14336/AD.2025.0409

**Published:** 2026-05-09

**Authors:** Wellydo Kesllowd Marinho Escarião, Guilherme Henrique Viana da Silva, Hellen Suzane Clemente de Castro, Sayonara Pereira da Silva, Nelyane Nayara Martins de Santana, Ramon Hypolito Lima, Felipe Porto Fiuza

**Affiliations:** ^1^Graduate Program in Neuroengineering, Edmond and Lily Safra International Institute of Neuroscience, Santos Dumont Institute, 59280-000, Macaíba, RN, Brazil.; ^2^Lemanic Neuroscience Doctoral School, Faculty of Psychology and Educational Sciences, University of Geneva, Geneva, Switzerland.; ^3^Center for the Interdisciplinary Study of Gerontology and Vulnerability, University of Geneva, Geneva, Switzerland.; ^4^Swiss Centre of Expertise in Life Course Research LIVES, University of Geneva, Geneva, Switzerland.; ^5^School of Anatomical Sciences, Faculty of Health Sciences, University of the Witwatersrand, Johannesburg, Republic of South Africa.

**Keywords:** Aging, Alzheimer’s Disease, Amyloid beta, Digital Pathology, Plaque-Associated microglia, Cognitive impairment

## Abstract

Alzheimer’s disease (AD), the most common form of dementia, is marked by cognitive decline and amyloid-beta (Aβ) plaque deposition in the brain. Microglia cluster around Aβ plaques shifting to a plaque-associated microglia (PAM) immunophenotype. This study investigates the association between Aβ, microglia and PAM with cognitive performance in 75 older adults, 39 with normal cognition and 36 with AD. Postmortem brain samples containing the middle temporal gyrus (MTG) underwent duplex immunohistochemistry for ionized calcium-binding adaptor molecule 1 (IBA1) and Aβ. A machine learning pipeline quantified parameters of Aβ, microglia and PAM expression. This study evaluated sex- and layer-specific patterns of expression of these parameters and the relationship with global cognitive performance, as measured by the Cognitive Abilities Screening Instrument (CASI) and the Mini-Mental State Examination (MMSE). Additionally, four specific cognitive domains, memory, executive function, language, and visuospatial processing, were evaluated. Aβ and PAM were significantly higher in AD, with no sex- or layer-specific differences. In layers 3 and 4, Aβ plaque size was inversely correlated with MMSE. In all layers, total and activated microglial densities were related to executive function, but in a cognitive status-dependent manner. In layers 5 and 6, higher PAM expression correlated with lower CASI and MMSE scores. PAM expressions in layers 3, 5, and 6 were negatively associated with memory scores. This study characterizes Aβ, microglia, and PAM patterns in the MTG, revealing layer-specific associations between histopathological metrics and cognitive domains. It also highlights PAM as a potential therapeutic target to mitigate cognitive decline in AD.

## INTRODUCTION

Dementia ranks as the seventh leading cause of death among diseases (World Health Organization, www.who.int/news-room/fact-sheets/detail/the-top-10-causes-of-death). Presently, it impacts over 55 million individuals across the globe, and projections suggest that by 2050, around 150 million people could be diagnosed with dementia-related conditions [1]. Alzheimer’s Disease (AD) is a progressive, age-related neurodegenerative disorder that accounts for roughly 60–80% of all diagnosed dementia cases [2]. In clinical settings, AD is most characterized by a memory-related cognitive decline [3]. Other symptoms include mood disturbances such as depression and anxiety, changes in sleep patterns, social withdrawal, hallucinations, delusions, and cognitive impairments affecting visual-spatial processing, language abilities, and executive functions [3–5]. Despite the growing burden of AD, there remains no cure, and current treatments offer only limited symptomatic relief, underscoring the urgent need for new therapeutic strategies [2].

The abnormal deposition of amyloid-beta (Aβ) plaques in the brain is a key pathological hallmark of AD [6]. Aβ is derived from the cleavage of amyloid precursor protein (APP), and under pathological conditions, it aggregates into insoluble plaques that accumulate in the extracellular space [7]. Amyloid deposits first form in the poorly myelinated regions of the basal neocortex, then gradually spread to adjacent areas and the hippocampus [6]. At later stages, Aβ plaques are found across the entire cerebral cortex, subcortical regions, brainstem and cerebellum [6]. The Aβ accumulation is thought to trigger a series of neurodegenerative processes, including tau hyperphosphorylation, oxidative stress, and neuroinflammation, all of which contribute to the clinical symptoms of AD [7].

The Aβ plaques can be detected decades before the onset of cognitive decline [8], [9]. By the age of 85 years, as many as 38% of cognitively unimpaired older adults may harbor Aβ deposits [10]. Aβ-targeted clinical trials have initially produced inconclusive results regarding their potential benefits for cognition [11]. Nonetheless, a recent meta-analysis including 16 randomized trials generated statistical evidence for a causal link between Aβ plaque reduction and decreased cognitive decline [12]. Moreover, 18 months of treatment with lecanemab, a humanized IgG1 monoclonal antibody targeting Aβ soluble protofibrils, reduced amyloid markers and attenuated the cognitive impairment among patients with early-stage AD [13], [14].

Neuroinflammatory processes underline the association between amyloid pathology and cognitive decline [15]. For instance, focal Aβ plaques, which are surrounded by inflammatory cells, are more strongly correlated with cognitive impairments than diffuse plaques [16]. Microglia, the primary immune cells of the central nervous system, play a crucial role in the Aβ-mediated neuroinflammation associated with AD [17], [18]. Upon detecting signals of injury or pathology, microglia transition into an activated state that is defined by retraction of their branched processes, enlarged soma, and higher phagocytic activity [19]. Microglial cells actively monitor the environment and phagocyte Aβ, preventing its accumulation [20]. Over time, there is insufficient clearance of Aβ and persistence of amyloid deposition, which leads to clustering of microglia around Aβ plaques [21]. These plaque-associated microglia (PAM) represent a specialized subset of microglia that show upregulation of genes related to lipid metabolism (e.g., ApoE, Lpl) and phagocytosis (Cst7, Trem2) while downregulating homeostatic microglial genes, such as Cx3cr1 and P2ry12 [22], [23].

The current literature points out that PAM may either mitigate or propagate damage depending on the stage of AD [24], [25]. However, data exploring the relationship between PAM and cognition, especially in humans, is still scarce. This paper aims to identify if there is an association between PAM expression and cognitive decline in AD. We analyzed several quantitative neuropathological metrics related to microglial, Aβ and PAM expression in the middle temporal gyrus (MTG). Like all neocortical regions, the MTG is structurally organized within six layers of neurons, each with distinct functions and connectivity [26–28]. Previous studies highlighted the need for data on region- and layer-specific histopathological alterations to better understand the selective vulnerability of neurons in normal aging and AD [29–33]. Thus, we also addressed if microglia, Aβ or PAM expression in specific cortical layers were associated with global cognitive performance measured by the Cognitive Abilities Screening Instrument (CASI) or the Mini Mental State Examination (MMSE). Finally, we evaluated if these histopathologic features influenced four specific cognitive domains, namely memory, executive function, language or visuospatial processing. Understanding how the MTG neuropathology affects cognitive performance could offer new insights into potential therapeutic strategies aimed at slowing or halting the AD clinical symptoms.

## MATERIALS AND METHODS

### Donors and brain samples

Data included in this study were obtained from the Seattle Alzheimer’s Disease Brain Cell Atlas (SEA-AD), an open database compiled by the Allen Institute for Brain Science, the University of Washington, and Kaiser Permanente Washington Research Institute (https://portal.brain-map.org/explore/seattle-alzheimers-disease). Major findings with this dataset were previously published by the database organizers [34] and other research groups [35]. The SEA-AD provides neuropathological, single-cell, and transcriptomic data from 84 participants, evenly divided between 42 individuals diagnosed with dementia and 42 without a dementia diagnosis, comprising 33 males and 51 females. The data were generated using postmortem brain tissue obtained from the Adult Changes in Thought (ACT) Study and the University of Washington Alzheimer’s Disease Research Center (UW ADRC). Upon enrollment, all participants consented to the brain donation. Comprehensive details on participant inclusion criteria, brain sample collection, and neurobiological procedures can be found in the documentation available on the database website (https://portal.brain-map.org/explore/seattle-alzheimers-disease/seattle-alzheimers-disease-brain-cell-atlasdownload?edit&amplanguage=enm).

The SEA-AD database provides de-identified information about the donors, such as age, sex, education years, and diagnosis based on the 4th edition of the Diagnostic and Statistical Manual of Mental Disorders [36]. It also includes global cognitive performance measurements from the most recent CASI and MMSE scores, as well as the interval, in months, between the administration of these assessments for each participant. Global metrics of AD pathology, such as Thal phase [37], CERAD [38], Braak [39], and AD Neuropathological Change (ADNC) [40] scores are also available. The database documentation also provides other metadata, such as the APOE genotype, brain pH, fresh brain weight, and postmortem interval (PMI).

Global cognitive functioning of participants enrolled in the ACT was assessed using the CASI and MMSE tools. Brain donors from the UW ADRC were evaluated only by the MMSE. The MMSE evaluates six cognitive domains, namely orientation, registration, attention and calculation, recall, language, and visual-constructional ability [41], [42]. The CASI evaluates nine cognitive domains, including long-term memory, short-term memory, mental manipulation, orientation, attention, abstraction and judgment, language skills, visual construction, and category fluency [43], [44]. The SEA-AD database also provides harmonized composite scores for four cognitive domains: memory (MEM), executive functioning (EXF), language (LAN), and visuospatial processing (VIS). To achieve this, database organizers assigned items from the neuropsychological batteries to one of the four domains. Bifactor models were then employed to psychometrically co-calibrate these domains across the cohorts. Cognitive domain-specific performance is reported in the database as z-scores, standardized against the AD population of the ACT study. Detailed information on the neuropsychological battery items and the statistical modeling used to evaluate each domain has been previously published by the database organizers [45].

In both the ACT and UW ADRC settings, a multidisciplinary consensus conference reviewed the neurological evaluation, detailed cognitive assessments, and information gathered from the individual and additional sources (such as a spouse, caregiver, family members, or others). Using this combined data, the team applied NIH research guidelines, such as the McKhann criteria, to reach a consensus diagnosis [46]. The diagnosis is listed in the database as “no dementia”, “AD”, “AD possible/probable”, other subtypes of dementia or unknown type of dementia. Using the clinical information combined with multiple neuropathological markers, the database organizers established a continuous pseudoprogression score (CPS) scale that represented the AD progression along a spectrum [34]. The CPS values range from 0 (minimal pathology) to 1 (severe pathology), and the changes associated with AD usually start at a CPS of 0.5 [34].

**Table 1.** Demographics, clinical and pathologic features of brain donors.

	Normal Cognition		Alzheimer’s Disease	
	Female	Male	Female	Male
N	23	16	23	13
Age (years)	90.3 ± 6.5	88.4 ± 8.6	87.4 ± 8.2	86.5 ± 9.7
Education (years)	15.5 ± 2.0	16.0 ± 2.7	15.9 ± 3.0	16.9 ± 2.6
PMI (hours)	7.0 ± 2.3	6.4 ± 2.2	6.5 ± 2.0	8.3 ± 2.0
APOE4 status (Carriers/Non-carriers)	5/18	3/13	8/15	5/8
ADNC score (Not AD/Low/Intermediate/High)	5/4/7/7	3/4/6/3	0/0/2/21	0/0/3/10
CPS score	0.49 ± 0.2	0.46 ± 0.2	0.81 ± 0.02	0.80 ± 0.11
CASI score	92.7 ± 6.1	92.9 ± 4.5	77.3 ± 6.6	81.9 ± 9.2
MMSE score	27.2 ± 2.6	26.1 ± 2.1	21.9 ± 2.9	23.6 ± 4.2

Continuous data are presented as mean ± SD.

*Abbreviations: ADNC* Alzheimer’s Disease Neuropathological Change, *APOE4* Alipoprotein-E4, *CASI* Cognitive Assessment Screening Instrument, *CPS* Continuous Pseudoprogression Score, *MMSE* Mini-Mental State Examination, *PMI* Postmortem interval.

From the 42 subjects classified as “no dementia” for cognitive status, 3 presented additional clinical notes suggesting mild cognitive impairments or multiple systems atrophy. For this reason, they were excluded from the present study. From now on, we refer to the remaining 39 individuals as the normal cognition (NC) group. Among the participants diagnosed with dementia (N = 42), all individuals diagnosed with definitive AD (N = 23) were included. Individuals diagnosed with AD possible/probable or unknown types of dementia were excluded if they either were classified as “Low” for the ADNC score (N = 4) or scored below 0.5 in the CPS (N = 2). Thus, our final sample consisted of 75 individuals, 39 with NC (23 female, 16 male) and 36 with AD (23 female, 13 male). Donors included in the present study ranged from 65 to 102 years (Table 1). In the supplemental material, we specify which donors of the SEAD database were included in the present study.

### Tissue processing

Methods for brain dissection, tissue processing, and image analysis were performed and described in detail by the database organizers [34], [47]. We will briefly summarize the procedures related to the data analyzed in the present study.

Firstly, blocks of brain tissue containing the MTG were sampled and embedded in paraffin. Then, brains were sliced into 5 µm sections and mounted into glass slides. One MTG section per individual was processed for the immunohistochemistry protocol. Sections were deparaffinized, immersed in xylene for 3 minutes at 3 times, rehydrated in a graded series of ethanol, and washed with tris-buffered saline with tween 20 (TBST). Following this, sections were submitted to a heat-induced epitope retrieval at 110°C for 15 minutes. An immuno-histochemistry (IHC) protocol was used to detect the Aβ clone 6E10 primary antibody (1:1,000, clone 6E10, mouse, cat. no. 803003, BioLegend). Then, sections were incubated with an anti-mouse secondary antibody, and the antigens were colorimetrically detected through horseradish peroxidase-mediated oxidation of 3,3′-diaminobenzidine (DAB, intelliPATH, cat. no. IPK5010), yielding a brown signal.

Following the DAB reaction, the sections were washed for 22 min in TBST and submitted to a duplex setup. For this step, sections were incubated with the primary antibody for the Ionized calcium binding adaptor molecule (IBA1), a marker of microglial cells (1:1,000, rabbit, cat. no. 019-19741, Wako). Then, sections were incubated with the species-matching secondary antibody conjugated with alkaline phosphatase. The final reaction was visualized with the intelliPATH Ferangi Blue Chromogen Kit (cat. no. IPK5027, Biocare Medical), which yielded a blue signal. After IHC, the sections were washed in TBST for 3 min, dehydrated in graded ethanol series, and cleared in xylene (or xylene substitute in the case of duplex IHC). Finally, the slides were coverslipped in a Tissue-Tek automated cover slipper (Sakura Finetek) using the Ecomount medium (cat. no. EM897L, Biocare Medical).

### Image analysis

Whole slide images were acquired at 20x magnification in an Aperio AT2 scanner (Leica Biosystems) and imported to a cloud-based server (Amazon Web Services). Several quality control measures were performed to assess positivity in the IHC slides, identify artifacts (e.g., non-specific DAB staining, bubbles or unstained areas), and detect features that could interfere with digital analysis, such as dark shadows, tissue folds, or tears. The images were then analyzed using the HALO v.3.4.2986 (Indica labs, Albuquerque, New Mexico, USA) software. Images from slides processed for IHC to detect neuronal nuclear protein (NeuN) were used to train a deep learning convolutional neural network for classifying cortical layers within the MTG. Thus, analysis was performed for the regions of interest (ROIs) defined as: entire gray matter (GM), Layer 1 (L1) - molecular layer, Layer 2 (L2) - external granular layer, Layer 3 (L3) - external pyramidal layer, Layer 4 (L4) - internal granular layer and Layers 5 and 6 (L5/6) - internal pyramidal and multiform layers.

**Figure 1.**
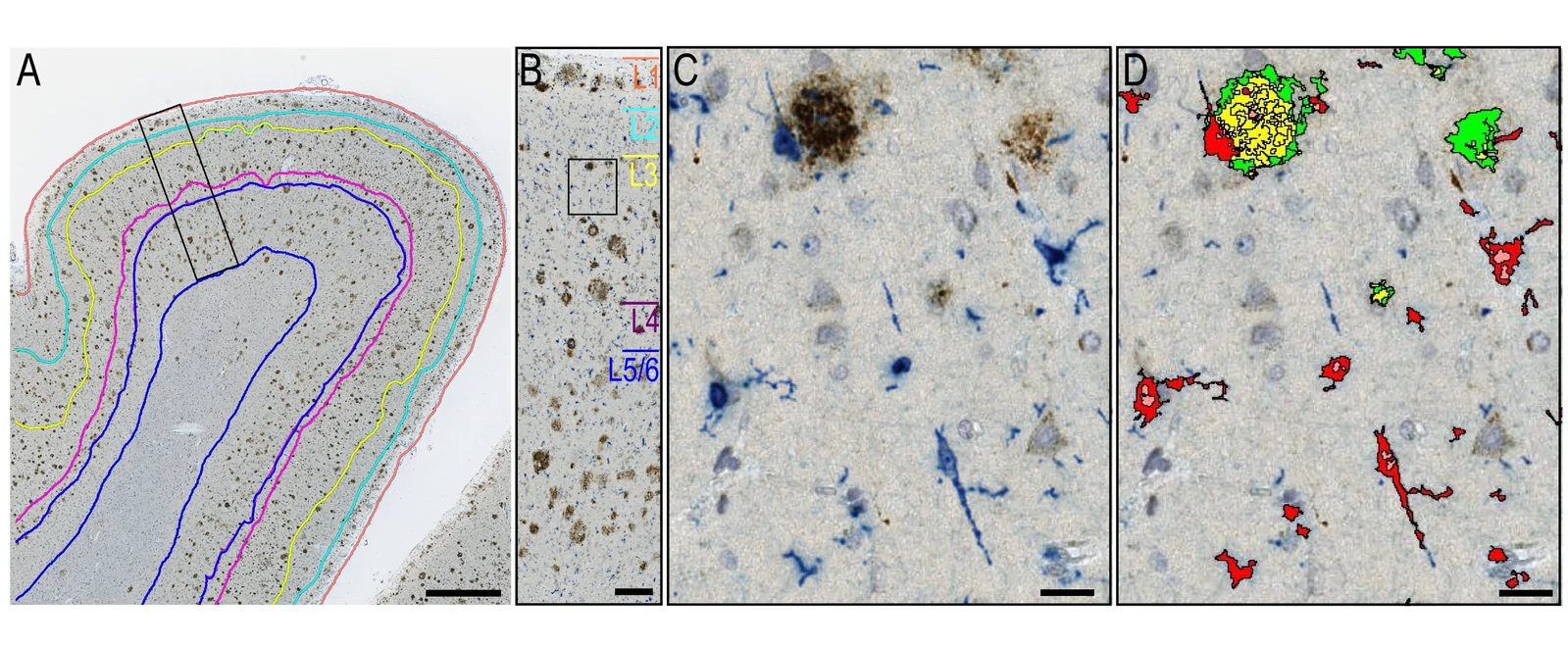
**Machine learning-based delimitation of middle temporal gyrus layers and histopathological features. (A)** Representative image of the middle temporal gyrus (MTG) subjected to duplex immunohistochemistry for Aβ (brown) and IBA1 (blue). A deep learning convolutional neural network was trained to delineate layer 1 (L1, orange), layer 2 (L2, cyan), layer 3 (L3, yellow), layer 4 (L4, purple), and layers 5 and 6 (L5/6, dark blue) of the MTG. **(B)** Higher-resolution view of the region outlined in A, illustrating MTG histopathology across the layers. **(C)** Higher-resolution view of the region outlined in B, highlighting Aβ plaques and IBA1-positive cells. **(D)** Machine learning pipeline outputs, identifying IBA1-positive cells (red), Aβ plaques (green), and the co-localization of IBA1-positive cell inclusions within Aβ plaques (yellow). Images and datasets were obtained from the Seattle Alzheimer’s Disease Brain Cell Atlas public database https://portal.brain-map.org/explore/seattle-alzheimers-disease. Scale bars: 1 mm (A), 150 µm (B), 20 µm (C-D).

The HALO software uses several modules for machine-learning based quantitative neuropathology. The HALO modules used for Aβ/IBA1 analyses were Color deconvolution, Analysis Quantification, Object colocalization and Microglia module. The modules are pre-trained with representative IBA1 and Aβ images to generate pseudo-colored markups labeling the detected microglial or Aβ objects. The microglia module is also trained to identify non-ramified IBA1+ cells with enlarged soma and retracted processes as morphologically activated. The object colocalization module outputs microglial cells as red markups, amyloid aggregates as green markups and the colocalization of microglial cell inclusions within amyloid plaques as yellow markups, which we refer here as PAM inclusions (Fig 1). In the primary study that generated the SEA-AD dataset [34] analyzed in the present work, quantification accuracy was validated by visual inspection of pseudo-colored overlays, ensuring fidelity between machine-identified objects and the underlying histology. These overlays were reviewed across all slides prior to analysis to confirm the reliability of cell and plaque detection.

The features extracted by the HALO modules that we analyzed here were: Aβ area fraction (percent of the MTG occupied by the Aβ staining), Aβ plaque area (average Aβ plaque area in μm^2^), Aβ plaque density (number of the Aβ objects divided by the MTG ROI in mm^2^), IBA1 density (number of the total IBA1 objects divided by the MTG ROI in mm^2^), Activated IBA1 density (number of the IBA1 objects classified as morphologically activated divided by the MTG ROI in mm^2^) and PAM inclusions (percent of IBA1 and Aβ positive colocalized objects). Detailed information of procedures for image analysis and acquisition of these morphometric parameters can be found in the documentation available on the database website (https://portal.brain-map.org/explore/seattle-alzheimers-disease/seattle-alzheimers-disease-brain-cell-atlas-download?edit&amplanguage=enm). The SEA-AD dataset also provides publicly accessible code via Jupyter Notebooks (available at: https://github.com/AllenInstitute/SEA-AD_2024/tree/main) and .csv files containing quantitative neuropathology metrics for each donor, which can be used to cross-validate analyses.

### Statistical analysis

First, we tested the hypothesis that AD-related cellular and pathological changes in the MTG could be expressed in a layer- and sex-specific manner. For this analysis, individuals were grouped by sex and further subdivided by cognitive status (e.g., female NC, female AD, male NC, male AD). We evaluated the distribution, asymmetries, and kurtosis of the Aβ area fraction, average Aβ plaque area, Aβ density, total IBA1 density, activated IBA1 density, and the percentage of PAM inclusions. Normality tests revealed a non-normal distribution for most of these histopathological variables. Therefore, within each sex-cognitive status subgroup, we used the Kruskal-Wallis test with Dunn’s post hoc to compare the histopathological parameter expression between cortical layers. Additionally, within each sex, we employed Mann-Whitney tests to compare differences in morphological parameter expression between cognitive status subgroups (e.g., female NC vs. female AD and male NC vs. male AD).

Next, we examined the hypothesis that layer-specific AD-related cellular and pathological changes in the MTG are associated with cognitive performance. For each ROI, linear regression models were used to test the association between morphological variables and global (CASI or MMSE) or domain-specific (memory, executive functioning, language and visuospatial processing) cognitive scores as the outcome variables. These models were adjusted for age, sex, APOE4 genotype, interval since the last cognitive test, diagnostic group, and postmortem interval. We then introduced an interaction term between the morphological variable and diagnostic group to assess whether the effects were specific to the AD or NC group. The underlying assumptions for multiple regressions (e.g., normality of residuals and homoscedasticity) were checked and validated for all models.

To visualize the multiple regression results, we z-transformed the predictors and fitted the models to obtain standardized coefficients. Forest plots were used to display these coefficients along with their 95% confidence intervals (CIs) for each neuropathological predictor in their respective model. The distance from zero represents the strength and direction of the association with cognitive scores, with negative associations to the left and positive associations to the right. The CI reflects the precision of the estimate, and p-values indicate statistical significance.

Statistical tests and graphs were performed either using GraphPad Prism version 10.0.0 (GraphPad Software, Boston, Massachusetts USA) or R (version 4.3.1) and R Studio (version 2024.12.1).

## RESULTS

We investigated whether the expression of Aβ, microglia or PAMs in the MTG exhibited layer- or sex-specific patterns. Firstly, we compared the morphological parameters across the cortical layers within each sex-cognitive status subgroup. We found no evidence for preferential accumulation of Aβ plaques, microgliosis or association of microglial cells with Aβ plaques in any MTG layer (Table 2).

**Table 2:** Sex- and Layer-specific evaluation of the MTG histopathology.

Variable	Sex	Cognitive Status	MTG ROI						Kruskal-Wallis (p-Value)
			GM	L1	L2	L3	L4	L5/6	
Aβ area fraction	Female	NC	1.42 (2.4)	1.27 (2.5)	1.14 (1.8)	1.83 (2.8)	1.47 (2.0)	1.23 (2.4)	0.91
		AD	3.09 (2.4)	2.33 (2.0)	2.97 (3.0)	3.96 (3.0)	3.41 (2.4)	2.6 (2.2)	0.77
	Mann-Whitney (p-Value)		0.0009	0.003	0.0009	0.002	0.001	0.001	
	Male	NC	1.2 (1.3)	0.82 (1.0)	0.94 (1.1)	1.48 (1.5)	1.23 (1.3)	1.06 (1.3)	0.17
		AD	4.50 (4.2)	4.25 (4.2)	5.27 (5.7)	6.16 (5.4)	4.98 (4.9)	2.79 (3.0)	0.28
	Mann-Whitney (p-Value)		0.005	0.002	0.002	0.002	0.006	0.025	
Average Aβ plaque area	Female	NC	205 (168)	182 (174)	221 (192)	205 (171)	199 (156)	205 (192)	0.98
		AD	315 (81)	270 (91.2)	341 (115)	298 (84.5)	297 (81.6)	337 (107)	0.50
	Mann-Whitney (p-Value)		0.007	0.038	0.014	0.024	0.011	0.006	
	Male	NC	212 (175)	120 (104)	187 (169)	184 (128)	184 (178)	225 (192)	0.13
		AD	337 (87.3)	268 (114)	342 (121)	305 (99.6)	346 (101)	390 (165)	0.13
	Mann-Whitney (p-Value)		0.027	0.001	0.011	0.01	0.007	
Aβ plaque density	Female	NC	45.5 (58.5)	47 (74.5)	36.7 (60.7)	57.6 (73.1)	51.4 (62.2)	39.4 (57.7)	0.90
		AD	100.4 (73.7)	91.5 (80)	84.1 (71.9)	130 (85.3)	115 (77.3)	81.7 (81.6)	0.94
	Mann-Whitney (p-Value)		0.002	0.003	0.0006	0.0009	0.001	0.008	
	Male	NC	45.7 (49.9)	49.4 (62.7)	39.5 (50.6)	59.7 (69.6)	52.6 (63.8)	29.4 (31.2)	0.06
		AD	147 (115)	160 (126)	160 (152)	207 (155)	151 (133)	87.3 (90.1)	0.18
	Mann-Whitney (p-Value)		0.009	0.006	0.006	0.005	0.011	0.022	
IBA1 density	Female	NC	72.1 (35.1)	75.1 (47.8)	77.67 (45.3)	72.02 (34.7)	65.49 (30.8)	70.60 (31.7)	0.75
		AD	89.1 (39)	85.1 (58.9)	101.8 (62.7)	91.05 (47.0)	76.74 (37.6)	78.44 (37.0)	0.91
	Mann-Whitney (p-Value)		0.17	0.50	0.08	0.11	0.22	0.25	
	Male	NC	88.7 (48.8)	94.72 (65.6)	88.66 (55.6)	81.73 (50.6)	75.07 (48.3)	80.99 (49.2)	0.57
		AD	82.2 (39.4)	75.72 (46.7)	83.02 (43.4)	(76.35) (43.7)	(70.24) (40.9)	76.26 (45.6)	0.92
	Mann-Whitney (p-Value)		0.78	0.47	0.99	0.95	0.92	0.82	
Activated IBA1 density	Female	NC	56.56 (29.3)	54.22 (37.8)	59.30 (34.0)	58.66 (30.2)	52.42 (29.2)	55.83 (27.0)	0.41
		AD	56.80 (41.5)	52.69 (49.1)	62.58 (54.3)	62.71 (47.1)	51.47 (38.1)	52.55 (37.0)	0.99
	Mann-Whitney (p-Value)		0.92	0.72	0.96	0.81	0.95	0.90	
	Male	NC	75.24 (51.5)	74.22 (51.6)	77.18 (56.2)	74.80 (52.3)	70.06 (49.9)	76.16 (50.8)	0.78
		AD	57.03 (45.2)	51.60 (46.1)	64.50 (48.9)	59.16 (46.6)	51.23 (40.1)	55,96 (45.4)	0.81
	Mann-Whitney (p-Value)		0.29	0.21	0.53	0.42	0.35	0.25	
PAM inclusions	Female	NC	5.41 (8.6)	5.26 (9.72)	4.48 (8.1)	6.92 (10.6)	6.36 (9.27)	4.6 (8.1)	0.90
		AD	16.7 (9.64)	13.5 (9.64)	12.7 (8.52)	20.5 (11)	20.9 (11.3)	14.9 (11.2)	0.88
	Mann-Whitney (p-Value)		<0.0001	<0.0001	0.0001	<0.0001	<0.0001	<0.0001	
	Male	NC	5.89 (7.83)	5.08 (7.3)	4.86 (6.49)	7.5 (10)	6.88 (8.86)	4.5 (6.97)	0.27
		AD	20.6 (15.3)	20 (15.3)	21.5 (18.8)	27.6 (20.2)	23.3 (18.3)	13 (11.7)	0.24
	Mann-Whitney (p-Value)		0.001	0.001	0.001	0.001	0.002	0.009	

Kruskal-Wallis tests were used to compare the histopathological variable between layers within each sex-cognitive status subgroup. Mann-Whitney tests were used to compare Female NC vs AD and Male NC vs AD groups. Data are presented as mean (SD). Significant p-values are highlighted in bold. *Abbreviations: AD* Alzheimer’s Disease, *GM* Gray Matter, *IBA1* Ionized calcium-binding adaptor molecule 1, *MTG* Middle Temporal Gyrus, *NC* Normal Cognition, *PAM* Plaque-associated microglia, *ROI* Region of Interest, *L* Layer.

*Abbreviations: AD* Alzheimer’s Disease, *GM* Gray Matter, *IBA1* Ionized calcium-binding adaptor molecule 1, *MTG* Middle Temporal Gyrus, *NC* Normal Cognition, *PAM* Plaque-associated microglia, *ROI* Region of Interest, *L* Layer.

**Figure 2.**
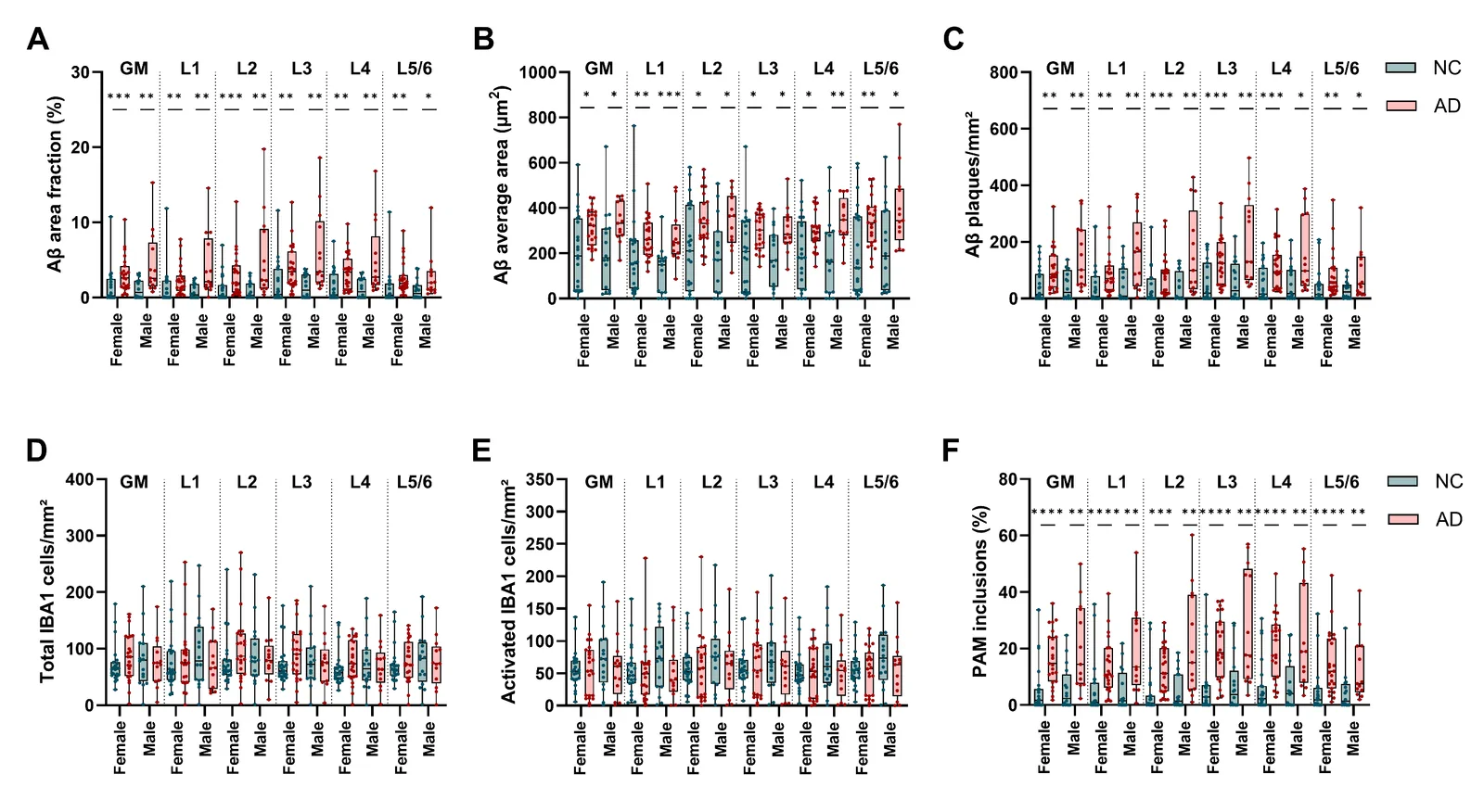
**Sex- and layer-specific expression of middle temporal gyrus histopathology.** Histopathological features of the middle temporal gyrus (MTG) were assessed in individuals with normal cognition (NC, green) and Alzheimer’s disease (AD, red). Box plots show the median and interquartile range for the following measures: Aβ area fraction **(A)**, average Aβ plaque area **(B)**, Aβ plaque density **(C)**, total IBA1-positive cell density **(D)**, activated IBA1-positive cell density **(E)**, and the percentage of plaque-associated microglia inclusions within Aβ plaques **(F).** The MTG was segmented into six regions of interest: entire gray matter (GM), layer 1 (L1), layer 2 (L2), layer 3 (L3), layer 4 (L4), and layers 5 and 6 (L5/6). Statistical significance for Mann-Whitney pairwise comparisons is indicated as * (p &lt 0.05), ** (p &lt 0.01), *** (p &lt 0.001), and **** (p &lt 0.0001).

Then, we compared the morphological parameters between individuals with NC and those with AD across each MTG layer and the entire GM of male and females. As anticipated, individuals with AD expressed significantly higher Aβ loads, average plaque areas, and densities compared to the NC group. This AD-related increase in amyloid plaques was consistent across all cortical layers. No sex differences in these parameters of amyloid expression were observed between the AD and NC groups (Table 2, Fig 2A-C). The total and activated microglial densities also did not differ between AD and NC groups across any sex or MTG layer (Table 2, Fig 2D-E). Similar to the Aβ findings, the percentage of PAM inclusions was significantly higher in AD individuals compared to the NC group. This pattern was reproduced across all cortical layers and we found no evidence for sex differences in AD-related PAM expression (Table 2, Fig 2F).

Multiple regression models were used to investigate associations between the histopathological features of the MTG with CASI and MMSE scores. The models were adjusted by age, sex, APOE4 genotype, interval from the last cognitive test, diagnostic group and postmortem interval. An interaction term between the diagnostic group and the morphological parameters was included to assess diagnostic-specific effects. The tables 3 and 4 summarizes the associations found between these variables.

**Figure 3.**
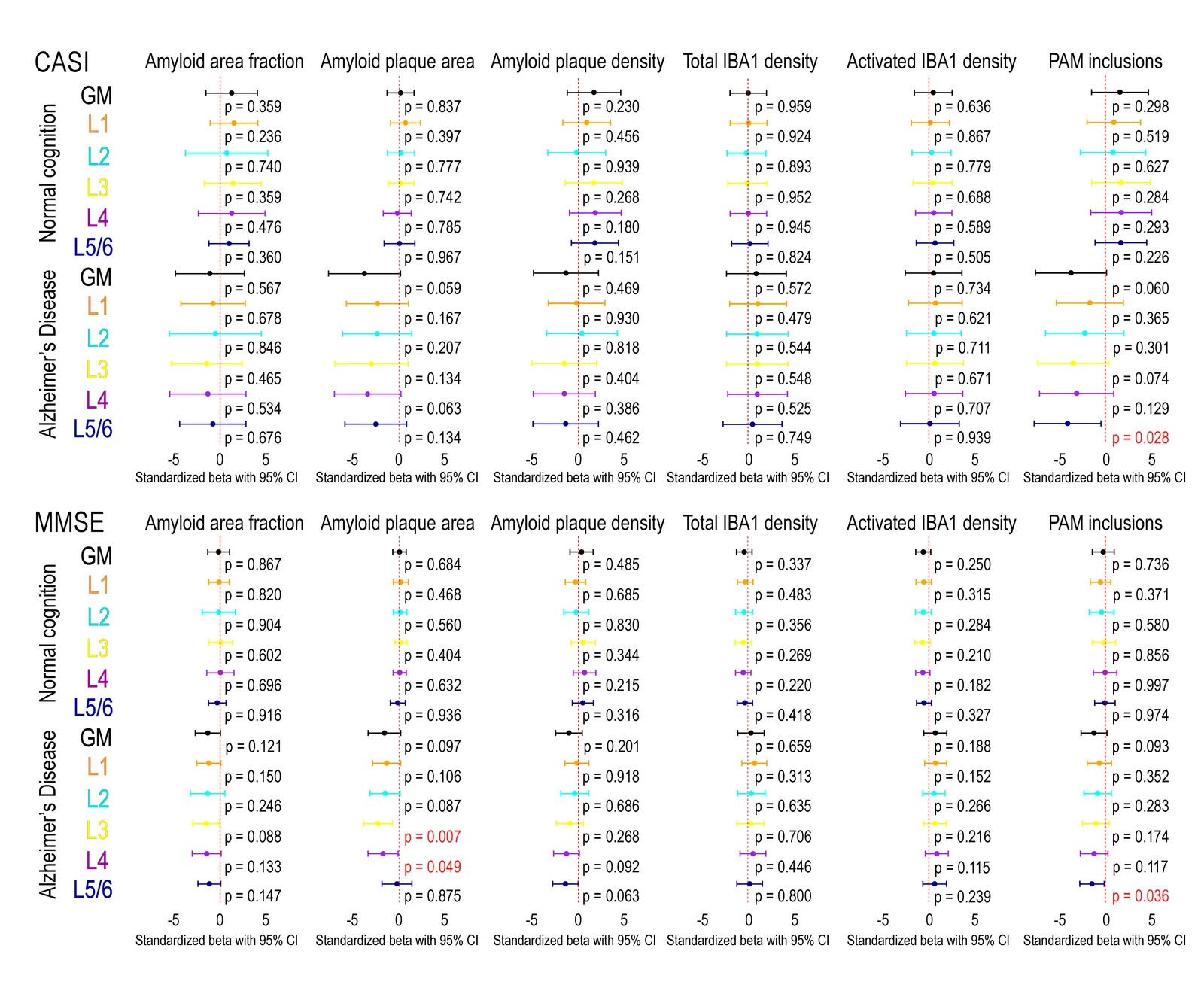
**Associations between middle temporal gyrus layer-specific histopathology and global cognition.** Multiple regression models were used to examine associations between middle temporal gyrus (MTG) histopathology and global cognitive scores assessed by the Cognitive Abilities Screening Instrument (CASI) and Mini-Mental State Examination (MMSE). Models were adjusted for age, sex, APOE4 genotype, time since the last cognitive test, diagnostic group, and postmortem interval. Forest plots display standardized slopes with 95% confidence intervals (CIs) for each neuropathological predictor. The distance from zero represents the strength and direction of the association, with negative associations to the left and positive associations to the right. CIs indicate the precision of the estimates, and p-values highlighted in red denote statistical significance.

Greater percentages of PAM inclusions in L5/6 were found to be associated with lower CASI scores for AD (βAD = -0.41, p = 0.028) but not NC individuals (βNC = 0.17, p = 0.23). This association was specific to this region, as it was not observed in GM, L1, L2, L3, or L4. CASI scores were not related to the Aβ area fraction, the average area of Aβ plaques, or the density of Aβ plaques across GM, L1, L2, L3, L4, or L5/6. Similarly, there was no relationship between CASI scores and total or activated microglial density in GM, L1, L2, L3, L4, or L5/6 (Table 3; Fig 3).

Similarly, greater percentages of PAM inclusions in L5/6 were associated with lower MMSE scores for AD (βAD = -0.14, p = 0.036) but not NC individuals (βNC =-0.002, p = 0.97). This association was also specific to this region, as it was not observed in GM, L1, L2, L3, or L4. MMSE scores were not related to the Aβ area fraction or the density of Aβ plaques across GM, L1, L2, L3, L4, or L5/6. However, higher average areas of Aβ plaques were associated with lower MMSE scores for AD individuals in L3 (βAD = -0.017, p = 0.007) and L4 (βAD = -0.012, p = 0.049). This association was not found in NC individuals. Finally, there was no relationship between MMSE scores and total or activated microglial density in GM, L1, L2, L3, L4, or L5/6 (Table 4; Fig 3).

**Table 3:** Associations between MTG histopathology and CASI scores.

Variable	ROI	Cognitive Status					
		NC			AD		
		β	SE	*p*	β	SE	*p*
Aβ area fraction	GM	0.541	0.58	0.359	-0.451	0.78	0.567
	L1	0.668	0.56	0.236	-0.316	0.76	0.678
	L2	0.262	0.78	0.74	-0.171	0.87	0.846
	L3	0.470	0.51	0.359	-0.465	0.63	0.465
	L4	0.514	0.72	0.476	-0.513	0.82	0.534
	L5/6	0.506	0.55	0.36	-0.380	0.90	0.676
Average Aβ plaque area	GM	0.001	0.01	0.837	-0.034	0.02	0.059
	L1	0.006	0.01	0.397	-0.020	0.02	0.167
	L2	0.002	0.01	0.777	-0.018	0.01	0.207
	L3	0.002	0.01	0.742	-0.027	0.02	0.134
	L4	-0.002	0.01	0.785	-0.031	0.02	0.063
	L5/6	0.000	0.01	0.967	-0.019	0.01	0.134
Aβ plaque density	GM	0.026	0.02	0.23	-0.019	0.03	0.469
	L1	0.013	0.02	0.456	-0.002	0.02	0.930
	L2	-0.002	0.02	0.939	0.006	0.03	0.818
	L3	0.020	0.02	0.268	-0.017	0.02	0.404
	L4	0.026	0.02	0.181	-0.020	0.02	0.386
	L5/6	0.032	0.02	0.151	-0.023	0.03	0.462
IBA1 density	GM	-0.003	0.03	0.959	0.058	0.05	0.572
	L1	0.000	0.02	0.924	0.041	0.03	0.479
	L2	-0.006	0.02	0.893	0.045	0.04	0.544
	L3	-0.006	0.03	0.952	0.057	0.04	0.548
	L4	-0.004	0.03	0.945	0.067	0.05	0.525
	L5/6	0.000	0.03	0.824	0.052	0.05	0.749
Activated IBA1 density	GM	0.010	0.03	0.636	0.045	0.04	0.734
	L1	0.003	0.03	0.867	0.034	0.04	0.621
	L2	0.005	0.03	0.779	0.031	0.04	0.711
	L3	0.007	0.03	0.688	0.041	0.04	0.671
	L4	0.010	0.03	0.589	0.047	0.05	0.707
	L5/6	0.013	0.03	0.505	0.035	0.05	0.939
PAM inclusions	GM	0.145	0.14	0.298	-0.332	0.17	0.060
	L1	0.085	0.13	0.519	-0.150	0.17	0.365
	L2	0.075	0.15	0.627	-0.194	0.19	0.301
	L3	0.122	0.11	0.284	-0.247	0.14	0.074
	L4	0.134	0.13	0.293	-0.237	0.15	0.129
	L5/6	0.172	0.14	0.226	-0.409	0.18	0.028

Multiple regression models were established with CASI scores as the outcome variable and histopathological parameters in the MTG ROI as explanatory variables. Models were adjusted for the covariates: cognitive status, age, sex, APOE4 status, postmortem interval and interval in months which the last CASI test was administered. An interaction term between cognitive status and the histopathological variable was used to assess cognitive status-dependent associations. Data are presented as the standardized β coefficient with standard error and adjusted p-values. Significant p-values are highlighted in bold.

*Abbreviations: AD* Alzheimer’s Disease,*GM* Gray Matter,*IBA1* Ionized calcium-binding adaptor molecule 1, *L* Layer, *MTG* Middle Temporal Gyrus, *NC* Normal Cognition, *PAM* Plaque-associated microglia, *ROI* Region of Interest, *SE* standard error.

**Figure 4.**
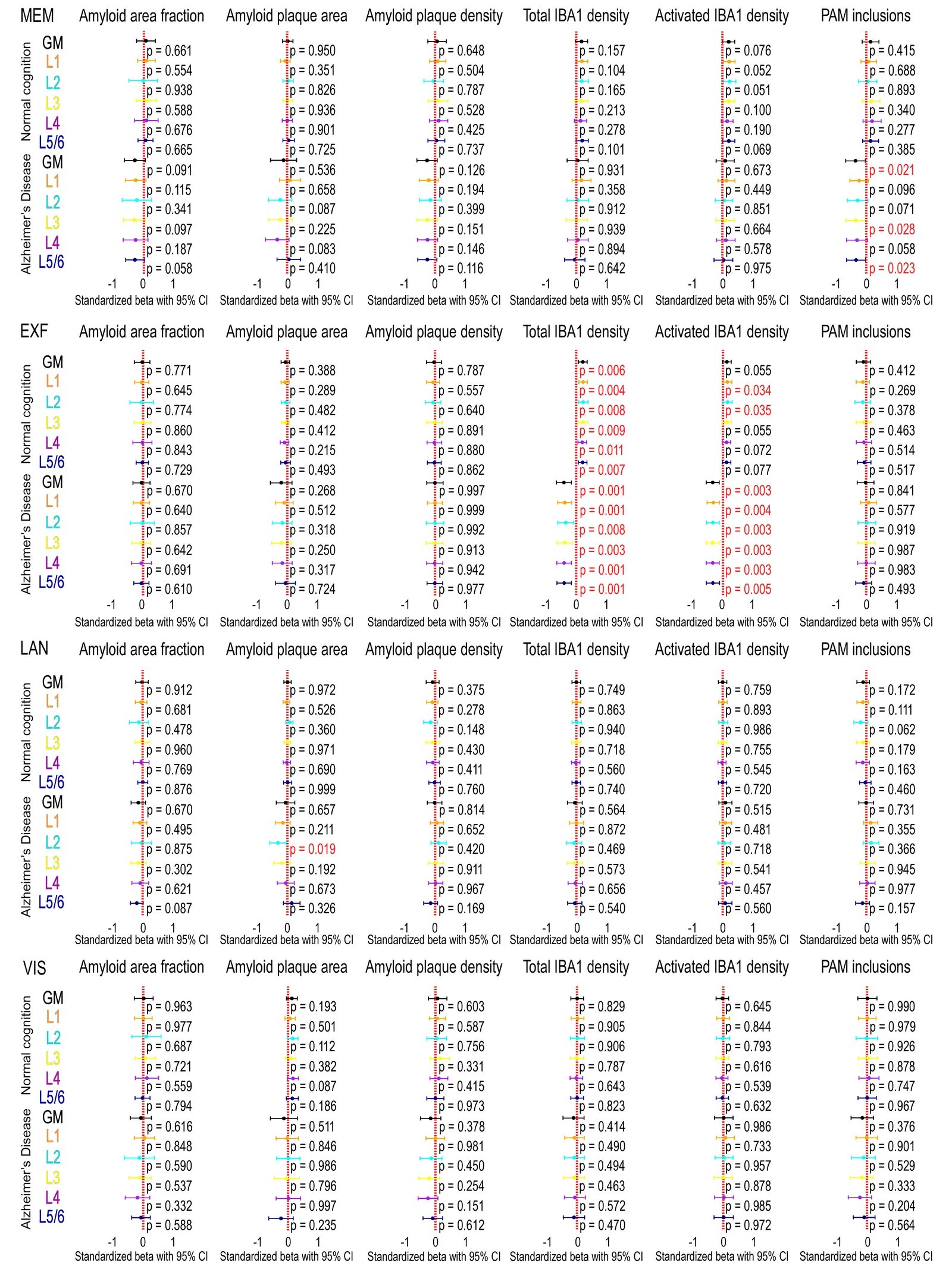
**Associations between middle temporal gyrus layer-specific histopathology and cognitive domains.** Multiple regression models were used to examine associations between middle temporal gyrus (MTG) histopathology and domain-specific cognitive scores. The cognitive domains assessed included memory (MEM), executive functioning (EXF), language (LAN), and visuospatial processing (VIS). Models were adjusted for age, sex, APOE4 genotype, diagnostic group, and postmortem interval. Forest plots display standardized slopes with 95% confidence intervals (CIs) for each neuropathological predictor. The distance from zero represents the strength and direction of the association, with negative associations to the left and positive associations to the right. CIs indicate the precision of the estimates, and p-values highlighted in red denote statistical significance.

The cognitive domain-specific analysis revealed that greater percentages of PAM inclusions in L5/6 were significantly associated with lower memory z-scores in AD individuals (βAD = -0.039, p = 0.023). Similar inverse associations were observed for percentages of PAM inclusions in L3 (βAD = -0.028, p = 0.028) and GM (βAD = -0.036, p = 0.021), but not in L4 (βAD = -0.027, p = 0.058), L2 (βAD = -0.029, p = 0.071), or L1 (βAD = -0.025, p = 0.096). No associations between memory performance and other morphological variables were detected in any layer (Table 5; Fig 4).

While microglial densities were not linked to global cognitive performance, significant correlations with executive function emerged (Table 6; Fig 4). In NC individuals, positive associations were observed between executive function z-scores and total microglial densities in GM (βNC = 0.006, p = 0.006), L1 (βNC = 0.005, p = 0.004), L2 (βNC = 0.005, p = 0.008), L3 (βNC = 0.006, p = 0.009), L4 (βNC = 0.006, p = 0.011), and L5/6 (βNC = 0.006, p = 0.007). In NC individuals, positive associations of executive function scores were also found with activated microglial densities, though only in L1 (βNC = 0.004, p = 0.034) and L2 (βNC = 0.004, p = 0.035).

In contrast, AD individuals exhibited negative associations between executive function z-scores and total microglial densities in GM (βAD = -0.01, p = 0.001), L1 (βAD = -0.009, p &lt 0.001), L2 (βAD = -0.01, p = 0.008), L3 (βAD = -0.01, p = 0.003), L4 (βAD = -0.01, p = 0.001), and L5/6 (βAD = -0.01, p = 0.001). Similarly, inverse correlations were noted with activated microglial densities in GM (βAD = -0.007, p = 0.003), L1 (βAD = -0.007, p = 0.004), L2 (βAD = -0.007, p = 0.003), L3 (βAD = -0.008, p = 0.003), L4 (βAD = -0.009, p = 0.003), and L5/6 (βAD = -0.008, p = 0.005). No other morphological variables were associated with executive function in any layer (Table 7; Fig 4).

Finally, analysis of language performance revealed that a higher average area of Aβ plaques was associated with lower z-scores for language in L2 of AD individuals (βAD = -0.002, p = 0.019). No additional associations with language were observed in any layer. No associations of visuospatial processing with the histopathological variables were observed in any MTG ROI (Table 8; Fig 4).

**Table 4:** Associations between MTG histopathology and MMSE scores.

Variable	ROI	Cognitive Status					
		NC			AD		
		β	SE	*p*	β	SE	*p*
Aβ area fraction	GM	0.040	0.23	0.867	-0.426	0.27	0.121
	L1	0.052	0.23	0.820	-0.391	0.27	0.150
	L2	0.036	0.30	0.904	-0.365	0.31	0.246
	L3	0.106	0.20	0.602	-0.392	0.23	0.088
	L4	0.109	0.28	0.696	-0.449	0.30	0.133
	L5/6	-0.200	0.17	0.916	-0.008	0.01	0.147
Average Aβ plaque area	GM	0.001	0.00	0.684	-0.012	0.01	0.097
	L1	0.002	0.00	0.468	-0.009	0.01	0.106
	L2	0.001	0.00	0.560	-0.009	0.01	0.087
	L3	0.002	0.00	0.404	-0.017	0.01	0.007
	L4	0.001	0.00	0.632	-0.012	0.01	0.049
	L5/6	0.000	0.00	0.936	-0.001	0.01	0.875
Aβ plaque density	GM	0.006	0.01	0.485	-0.013	0.01	0.201
	L1	-0.003	0.01	0.685	-0.001	0.01	0.918
	L2	-0.002	0.01	0.830	-0.004	0.01	0.687
	L3	0.007	0.01	0.344	-0.009	0.01	0.268
	L4	0.010	0.01	0.215	-0.015	0.01	0.092
	L5/6	0.009	0.01	0.316	-0.021	0.01	0.063
IBA1 density	GM	-0.012	0.01	0.337	0.009	0.02	0.659
	L1	-0.006	0.01	0.483	0.015	0.01	0.313
	L2	-0.010	0.01	0.356	0.008	0.02	0.635
	L3	-0.014	0.01	0.269	0.008	0.02	0.706
	L4	-0.016	0.01	0.220	0.017	0.02	0.446
	L5/6	-0.010	0.01	0.418	0.005	0.02	0.800
Activated IBA1 density	GM	-0.014	0.01	0.250	0.025	0.02	0.188
	L1	-0.011	0.01	0.315	0.023	0.02	0.152
	L2	-0.012	0.01	0.284	0.017	0.02	0.266
	L3	-0.015	0.01	0.210	0.022	0.02	0.216
	L4	-0.017	0.01	0.182	0.032	0.02	0.115
	L5/6	-0.012	0.01	0.327	0.024	0.02	0.239
PAM inclusions	GM	-0.018	0.05	0.736	-0.110	0.06	0.093
	L1	-0.046	0.05	0.371	-0.057	0.06	0.352
	L2	-0.033	0.06	0.580	-0.072	0.07	0.283
	L3	-0.008	0.05	0.856	-0.072	0.05	0.174
	L4	0.000	0.05	0.997	-0.093	0.06	0.117
	L5/6	-0.002	0.06	0.974	-0.145	0.07	0.036

Multiple regression models were established with MMSE scores as the outcome variable and histopathological parameters in the MTG ROI as explanatory variables. Models were adjusted for the covariates: cognitive status, age, sex, APOE4 status, postmortem interval and interval in months which the last MMSE test was administered. An interaction term between cognitive status and the histopathological variable was used to assess cognitive status-dependent associations. Data are presented as the standardized β coefficient with standard error and adjusted p-values. Significant p-values are highlighted in bold.

*Abbreviations: AD* Alzheimer’s Disease, *GM* Gray Matter, *IBA1* Ionized calcium-binding adaptor molecule 1, *L* Layer, *MTG* Middle Temporal Gyrus, *NC* Normal Cognition, *PAM* Plaque-associated microglia, *ROI* Region of Interest, *SE* standard error.

## DISCUSSION

This study provides a comprehensive analysis of MTG histopathology and its association with cognitive performance in older adults with normal cognition or Alzheimer’s disease. The findings reveal AD-related alterations in the MTG, including increased amyloidosis and a higher association of microglial cells with Aβ plaques, with no changes in total or activated microglial densities. These findings were consistent across cortical layers and sexes. Most pathological changes were not directly associated with global cognitive scores, with the exception of average Aβ plaque areas in layers 3–4 and percentages of PAM inclusions in layers 5–6. However, analysis of specific cognitive domains revealed associations between Aβ, PAM, and microglial expression that were not apparent in the global scores.

**Table 5:** Associations between MTG histopathology and memory performance.

Variable	ROI	Cognitive Status					
		NC			AD		
		β	SE	*p*	β	SE	*p*
Aβ area fraction	GM	0.025	0.06	0.661	-0.109	0.06	0.091
	L1	0.033	0.05	0.554	-0.102	0.06	0.115
	L2	-0.006	0.07	0.938	-0.071	0.07	0.341
	L3	0.026	0.05	0.588	-0.089	0.05	0.097
	L4	0.028	0.07	0.676	-0.094	0.07	0.187
	L5/6	-0.049	0.04	0.665	0.000	0.00	0.058
Average Aβ plaque area	GM	0.000	0.00	0.950	-0.001	0.00	0.536
	L1	-0.001	0.00	0.351	0.001	0.00	0.658
	L2	0.000	0.00	0.826	-0.002	0.00	0.087
	L3	0.000	0.00	0.936	-0.002	0.00	0.225
	L4	0.000	0.00	0.901	-0.003	0.00	0.083
	L5/6	0.000	0.00	0.725	0.000	0.00	0.410
Aβ plaque density	GM	0.001	0.00	0.648	-0.004	0.00	0.126
	L1	0.001	0.00	0.505	-0.003	0.00	0.194
	L2	-0.001	0.00	0.787	-0.002	0.00	0.399
	L3	0.001	0.00	0.528	-0.003	0.00	0.151
	L4	0.002	0.00	0.425	-0.003	0.00	0.146
	L5/6	0.001	0.00	0.737	-0.004	0.00	0.116
IBA1 density	GM	0.004	0.00	0.157	0.000	0.00	0.931
	L1	0.003	0.00	0.104	0.003	0.00	0.358
	L2	0.003	0.00	0.165	0.000	0.00	0.912
	L3	0.004	0.00	0.213	0.000	0.00	0.939
	L4	0.003	0.00	0.278	0.001	0.01	0.894
	L5/6	0.005	0.00	0.101	-0.002	0.01	0.642
Activated IBA1 density	GM	0.005	0.00	0.076	0.002	0.00	0.673
	L1	0.005	0.00	0.052	0.003	0.00	0.449
	L2	0.005	0.00	0.051	0.001	0.00	0.851
	L3	0.005	0.00	0.100	0.002	0.00	0.664
	L4	0.004	0.00	0.190	0.003	0.00	0.578
	L5/6	0.005	0.00	0.069	0.000	0.00	0.975
PAM inclusions	GM	0.011	0.01	0.415	-0.036	0.02	0.021
	L1	0.005	0.01	0.688	-0.025	0.01	0.096
	L2	0.002	0.01	0.893	-0.029	0.02	0.071
	L3	0.010	0.01	0.340	-0.028	0.01	0.028
	L4	0.013	0.01	0.277	-0.027	0.01	0.058
	L5/6	0.012	0.01	0.385	-0.039	0.02	0.023

Multiple regression models were established with psychometrically co-calibrated memory z-scores as the outcome variable and histopathological parameters in the MTG ROI as explanatory variables. Models were adjusted for the covariates: cognitive status, age, sex, APOE4 status and postmortem interval. An interaction term between cognitive status and the histopathological variable was used to assess cognitive status-dependent associations. Data are presented as the standardized β coefficient with standard error and adjusted p-values. Significant p-values are highlighted in bold.

*Abbreviations: AD* Alzheimer’s Disease, *GM* Gray Matter, *IBA1* Ionized calcium-binding adaptor molecule 1, *L* Layer, *MTG* Middle Temporal Gyrus, *NC* Normal Cognition, *PAM* Plaque-associated microglia, *ROI* Region of Interest, *SE* standard error.

The MTG plays a crucial role in the perception of moving objects within the visual field [48] and is involved in semantic memory processing, such as mapping words and gestures into meaningful representations [49]. It appears to be a uniquely specialized region of the human brain, lacking a homologous structure in other animals [50]. Additionally, the MTG is critical for understanding neurodegeneration, as it is highly sensitive to morphological changes during the transition from the preclinical to clinical stages of AD [33], [34], [39].

Previous studies have highlighted the need for data on layer-specific alterations in brain regions like the MTG to better comprehend the selective vulnerability of neuronal subpopulations to AD [29–33]. The cerebral cortex typically exhibits higher Aβ deposition in layers 3 and 5, while layers 2 and 6 are relatively spared [6]. However, this pattern is region-specific; for example, in the frontal cortex, layers 2 and 3 show greater Aβ loads [51]. A spatial transcriptomic analysis suggested that layers 2–5 of the MTG may be particularly vulnerable to Aβ pathology, although this was not directly evaluated [33]. In the present study, we found no evidence of increased Aβ deposition in specific layers of the MTG. However, across all cortical layers, individuals with AD exhibited higher Aβ loads, average plaque areas, and Aβ densities compared to those with NC. This finding further suggests that layer-specific amyloidosis varies across brain regions and is not a uniform feature of the cerebral cortex.

**Table 6:** Associations between MTG histopathology and executive function.

Variable	ROI	Cognitive Status					
		NC			AD		
		β	SE	*p*	β	SE	*p*
Aβ area fraction	GM	-0.016	0.053	0.771	-0.026	0.060	0.670
	L1	-0.023	0.051	0.645	-0.028	0.059	0.640
	L2	-0.020	0.069	0.774	-0.013	0.070	0.857
	L3	-0.008	0.046	0.860	-0.023	0.050	0.642
	L4	-0.012	0.063	0.843	-0.026	0.066	0.691
	L5/6	-0.023	0.036	0.729	-0.002	0.001	0.610
Average Aβ plaque area	GM	-0.001	0.001	0.388	-0.002	0.001	0.268
	L1	-0.001	0.001	0.289	-0.001	0.001	0.512
	L2	0.000	0.001	0.482	-0.001	0.001	0.318
	L3	0.000	0.001	0.412	-0.002	0.001	0.250
	L4	-0.001	0.001	0.215	-0.001	0.001	0.317
	L5/6	0.000	0.001	0.493	0.000	0.001	0.724
Aβ plaque density	GM	-0.001	0.002	0.787	0.000	0.002	0.997
	L1	-0.001	0.002	0.557	0.000	0.002	0.999
	L2	-0.001	0.002	0.640	0.000	0.002	0.992
	L3	0.000	0.002	0.891	0.000	0.002	0.913
	L4	0.000	0.002	0.880	0.000	0.002	0.942
	L5/6	0.000	0.002	0.862	0.000	0.002	0.977
IBA1 density	GM	0.006	0.002	0.006	-0.014	0.004	0.001
	L1	0.005	0.002	0.004	-0.0094	0.003	0.001
	L2	0.005	0.002	0.008	-0.010	0.003	0.008
	L3	0.006	0.002	0.009	-0.013	0.004	0.003
	L4	0.006	0.002	0.011	-0.014	0.004	0.001
	L5/6	0.006	0.002	0.007	-0.014	0.004	0.001
Activated IBA1 density	GM	0.004	0.002	0.055	-0.008	0.004	0.003
	L1	0.004	0.002	0.034	-0.007	0.003	0.004
	L2	0.004	0.002	0.035	-0.007	0.003	0.003
	L3	0.004	0.002	0.055	-0.008	0.003	0.003
	L4	0.004	0.002	0.072	-0.009	0.004	0.003
	L5/6	0.004	0.002	0.077	-0.008	0.004	0.005
PAM inclusions	GM	-0.010	0.013	0.412	-0.003	0.015	0.841
	L1	-0.013	0.012	0.269	0.008	0.014	0.577
	L2	-0.012	0.014	0.378	0.002	0.015	0.919
	L3	-0.008	0.010	0.463	0.000	0.012	0.987
	L4	-0.007	0.011	0.514	0.000	0.013	0.983
	L5/6	-0.009	0.013	0.517	-0.011	0.016	0.493

Multiple regression models were established with psychometrically co-calibrated executive function z-scores as the outcome variable and histopathological parameters in the MTG ROI as explanatory variables. Models were adjusted for the covariates: cognitive status, age, sex, APOE4 status and postmortem interval. An interaction term between cognitive status and the histopathological variable was used to assess cognitive status-dependent associations. Data are presented as the standardized β coefficient with standard error and adjusted p-values. Significant p-values are highlighted in bold.

*Abbreviations: AD* Alzheimer’s Disease, *GM* Gray Matter, *IBA1* Ionized calcium-binding adaptor molecule 1, *L* Layer, *MTG* Middle Temporal Gyrus, *NC* Normal Cognition, *PAM* Plaque-associated microglia, *ROI* Region of Interest, *SE* standard error.

The prevalence of Alzheimer's disease (AD) is higher in women than in men, even after accounting for women's longer lifespans [52], [53]. This observation has sparked significant interest in understanding sex differences in the neuropathological features of AD [54]. Studies using animal models of Aβ overexpression have reported increased Aβ loads in females compared to males, particularly in regions such as the hippocampus and prefrontal cortex [55]. Similarly, a genome-wide association study identified a strong positive correlation between amyloidosis in the prefrontal cortex of women and serine protease inhibitor genes (SERPINB1, SERPINB6, and SERPINB9), with no such association observed in men [56]. Investigations using Aβ positron emission tomography (PET) imaging have yielded mixed results, with some studies finding no sex differences [57–59], while others reported higher Aβ levels in women [60], [61].

**Table 7:** Associations between MTG histopathology and language.

Variable	ROI	Cognitive Status					
		NC			AD		
		β	SE	*p*	β	SE	*p*
Aβ area fraction	GM	-0.0047	0.0424	0.912	-0.0551	0.0487	0.670
	L1	-0.0169	0.0408	0.681	-0.0331	0.0481	0.495
	L2	-0.0388	0.0544	0.478	-0.0089	0.0563	0.875
	L3	-0.0019	0.0364	0.960	-0.0423	0.0407	0.302
	L4	-0.0149	0.0505	0.769	-0.0267	0.0538	0.621
	L5/6	-0.0417	0.0306	0.876	-0.0001	0.0012	0.087
Average Aβ plaque area	GM	0.0000	0.0005	0.972	-0.0006	0.0012	0.657
	L1	-0.0003	0.0004	0.526	-0.0012	0.0009	0.211
	L2	0.0004	0.0004	0.360	-0.0022	0.0009	0.019
	L3	0.0000	0.0005	0.971	-0.0015	0.0011	0.192
	L4	-0.0002	0.0005	0.690	-0.0005	0.0011	0.673
	L5/6	0.0000	0.0004	0.999	0.0009	0.0009	0.326
Aβ plaque density	GM	-0.0014	0.0016	0.375	-0.0004	0.0018	0.814
	L1	-0.0013	0.0012	0.278	0.0006	0.0014	0.652
	L2	-0.0021	0.0014	0.148	0.0013	0.0016	0.420
	L3	-0.0010	0.0013	0.430	0.0002	0.0014	0.911
	L4	-0.0012	0.0014	0.411	0.0001	0.0016	0.967
	L5/6	-0.0005	0.0016	0.760	-0.0027	0.0020	0.169
IBA1 density	GM	-0.0007	0.0021	0.749	-0.0021	0.0036	0.564
	L1	-0.0003	0.0016	0.863	-0.0004	0.0026	0.872
	L2	-0.0001	0.0018	0.940	-0.0022	0.0030	0.469
	L3	-0.0008	0.0022	0.718	-0.0020	0.0035	0.573
	L4	-0.0014	0.0023	0.560	-0.0018	0.0039	0.656
	L5/6	-0.0007	0.0023	0.740	-0.0023	0.0038	0.540
Activated IBA1 density	GM	-0.0007	0.0022	0.759	0.0022	0.0034	0.515
	L1	-0.0003	0.0020	0.893	0.0020	0.0028	0.481
	L2	0.0000	0.0020	0.986	0.0010	0.0028	0.718
	L3	-0.0006	0.0021	0.755	0.0020	0.0032	0.541
	L4	-0.0013	0.0022	0.545	0.0027	0.0036	0.457
	L5/6	-0.0008	0.0022	0.720	0.0021	0.0036	0.560
PAM inclusions	GM	-0.0136	0.0098	0.172	-0.0040	0.0116	0.731
	L1	-0.0150	0.0093	0.111	0.0105	0.0112	0.355
	L2	-0.0204	0.0108	0.062	0.0110	0.0121	0.366
	L3	-0.0110	0.0081	0.179	0.0006	0.0094	0.945
	L4	-0.0126	0.0089	0.163	-0.0003	0.0104	0.977
	L5/6	-0.0075	0.0101	0.460	-0.0174	0.0121	0.157

Multiple regression models were established with psychometrically co-calibrated language z-scores as the outcome variable and histopathological parameters in the MTG ROI as explanatory variables. Models were adjusted for the covariates: cognitive status, age, sex, APOE4 status and postmortem interval. An interaction term between cognitive status and the histopathological variable was used to assess cognitive status-dependent associations. Data are presented as the standardized β coefficient with standard error and adjusted p-values. Significant p-values are highlighted in bold.

*Abbreviations: AD* Alzheimer’s Disease, *GM* Gray Matter, *IBA1* Ionized calcium-binding adaptor molecule 1, *L* Layer, *MTG* Middle Temporal Gyrus, *NC* Normal Cognition, *PAM* Plaque-associated microglia, *ROI* Region of Interest, *SE* standard error.

In the present study, we found no sex differences in AD-associated Aβ deposition within the MTG. Neuropathological evaluations of human samples have shown that females are more affected by global AD pathology, though this is primarily driven by neurofibrillary tangles [62], [63]. A previous study examining eight regions reported that AD women presented elevated neurofibrillary tangles in all ROIs, while Aβ pathology was significantly higher in only five areas: the inferior temporal cortex, hippocampus, entorhinal cortex, agranular gyrus, and calcarine cortex [62]. Despite these regional differences, the overall Aβ pathology showed no significant sex difference [62]. Additionally, an AD-related increase in Aβ load was observed in the parietal cortex exclusively in females, whereas no such increase was found in the temporal cortex [64]. Therefore, sex differences in amyloid expression appear to be highly region-dependent. Our findings suggest that the MTG may be one such region where these differences are minimal or absent.

**Table 8:** Associations between MTG histopathology and visuospatial processing.

Variable	ROI	Cognitive Status					
		NC			AD		
		β	SE	*p*	β	SE	*p*
Aβ area fraction	GM	0.0030	0.0646	0.963	-0.0365	0.0724	0.616
	L1	-0.0018	0.0620	0.977	0.0140	0.0724	0.848
	L2	0.0339	0.0836	0.687	-0.0462	0.0854	0.590
	L3	0.0198	0.0553	0.721	-0.0375	0.0605	0.537
	L4	0.0445	0.0757	0.559	-0.0780	0.0797	0.332
	L5/6	-0.0416	0.0437	0.794	-0.0001	0.0017	0.588
Average Aβ plaque area	GM	0.0010	0.0007	0.193	-0.0012	0.0018	0.511
	L1	0.0004	0.0007	0.501	-0.0003	0.0014	0.846
	L2	0.0010	0.0006	0.112	0.0000	0.0013	0.986
	L3	0.0006	0.0007	0.382	-0.0004	0.0017	0.796
	L4	0.0012	0.0007	0.087	0.0000	0.0016	0.997
	L5/6	0.0009	0.0007	0.186	-0.0016	0.0013	0.235
Aβ plaque density	GM	0.0012	0.0024	0.603	-0.0023	0.0026	0.378
	L1	0.0010	0.0018	0.587	0.0001	0.0021	0.981
	L2	0.0007	0.0022	0.756	-0.0018	0.0024	0.450
	L3	0.0018	0.0019	0.331	-0.0023	0.0020	0.254
	L4	0.0017	0.0021	0.415	-0.0033	0.0023	0.151
	L5/6	0.0001	0.0025	0.973	-0.0015	0.0029	0.612
IBA1 density	GM	-0.0007	0.0031	0.829	-0.0043	0.0052	0.414
	L1	-0.0003	0.0024	0.905	-0.0025	0.0036	0.490
	L2	-0.0003	0.0027	0.906	-0.0027	0.0039	0.494
	L3	-0.0009	0.0032	0.787	-0.0037	0.0050	0.463
	L4	-0.0016	0.0034	0.643	-0.0032	0.0057	0.572
	L5/6	-0.0008	0.0034	0.823	-0.0041	0.0056	0.470
Activated IBA1 density	GM	-0.0015	0.0033	0.645	-0.0001	0.0048	0.986
	L1	-0.0006	0.0029	0.844	0.0014	0.0042	0.733
	L2	-0.0008	0.0029	0.793	0.0002	0.0040	0.957
	L3	-0.0016	0.0032	0.616	-0.0007	0.0046	0.878
	L4	-0.0020	0.0033	0.539	0.0001	0.0051	0.985
	L5/6	-0.0016	0.0033	0.632	-0.0002	0.0052	0.972
PAM inclusions	GM	0.0002	0.0151	0.990	-0.0158	0.0178	0.376
	L1	-0.0004	0.0141	0.979	-0.0021	0.0170	0.901
	L2	-0.0015	0.0165	0.926	-0.0116	0.0184	0.529
	L3	0.0019	0.0122	0.878	-0.0138	0.0142	0.333
	L4	0.0044	0.0135	0.747	-0.0201	0.0157	0.204
	L5/6	-0.0007	0.0162	0.967	-0.0111	0.0192	0.564

Multiple regression models were established with psychometrically co-calibrated visuospatial processing z-scores as the outcome variable and histopathological parameters in the MTG ROI as explanatory variables. Models were adjusted for the covariates: cognitive status, age, sex, APOE4 status and postmortem interval. An interaction term between cognitive status and the histopathological variable was used to assess cognitive status-dependent associations. Data are presented as the standardized β coefficient with standard error and adjusted p-values. Significant p-values are highlighted in bold.

*Abbreviations: AD* Alzheimer’s Disease, *GM* Gray Matter, *IBA1* Ionized calcium-binding adaptor molecule 1, *L* Layer, *MTG* Middle Temporal Gyrus, *NC* Normal Cognition, *PAM* Plaque-associated microglia, *ROI* Region of Interest, *SE* standard error.

The relationship between Aβ deposition and cognitive changes in AD remains a topic of debate in the literature [65]. A recent PET study demonstrated that baseline Aβ levels predicted the rate of change in cognitive composite scores [66]. However, a systematic review reported that 10 out of 17 studies using PET or CSF-based analyses found no association between Aβ and cognition [67]. Notably, one study observed an association between Aβ and objective cognitive decline but not with subjective cognitive decline [68]. Postmortem studies have also shown mixed results, with some reporting no significant association between Aβ plaques and cognitive decline [69], [70], while others found negative correlations between these variables [16], [71].

These conflicting findings may be attributed to several factors. One possibility is that amyloidosis in specific brain regions plays a critical role in cognitive impairment. Alternatively, Aβ may selectively affect certain cognitive domains, and the variability in cognitive assessment methods across studies makes comparisons challenging. In the present study, no associations were found between Aβ loads or densities and CASI or MMSE scores in any layers of the MTG. However, the average area of Aβ plaques was negatively associated with MMSE scores in layers 3 and 4, with no such relationship observed for CASI scores. While both tests measure global cognition, the MMSE evaluates six cognitive domains, whereas the CASI assesses nine [16]. The difference found in the present study suggests that the broader assessment of the CASI may mask these associations by aggregating more information.

Recent studies have shown that impairments in specific cognitive domains, such as orientation, recall, and language, have a profound impact on cognitive decline, whereas others, like registration, exert minimal influence [72]. Even considering the four specific cognitive domains evaluated here, Aβ loads and densities were not associated with cognitive performance. Furthermore, the association between average Aβ plaque area in layers 3–4 and MMSE scores was not evident in any cognitive domain. However, in the MTG L2, a negative correlation was identified between Aβ plaque size and language scores. This result suggests that the growth of amyloid plaques in L2 may disrupt the local processing network, impairing the MTG role in language function. In summary, these findings indicate that the size of Aβ plaques, rather than their quantity, in the upper MTG layers plays a critical role in cognitive decline, particularly in language deficits.

Microgliosis is thought to be a common feature of many neurodegenerative diseases [18], [72]. For instance, in a triple transgenic mouse model of AD, higher densities of total and activated microglial cells were observed in the CA1 hippocampal region compared to control mice [73]. However, several human post-mortem studies have not found evidence of increased total microglial densities in AD [74–77]. In fact, a stereological study found a reduction in absolute microglial numbers in layers 1–2 and 5–6 of the entorhinal cortex in AD subjects [77]. Design-based stereology accounts for potential biases caused by the 3D organization of microglial cells and provides absolute estimates of cell numbers [78]. Although the present study corroborates the previous findings of no significant AD-related changes in microglial densities, the results would be more accurate if a stereological methodology was employed.

The microglial expression is known to differ between males and females in a region- and age-specific manner [79–82]. For example, in 3-week-old wild-type mice, IBA1 density is higher in the hippocampus and lower in the amygdala of males compared to females, whereas in 13-week-old male mice, IBA1 density is elevated in the hippocampus, cortex, and amygdala [79]. Additionally, transcriptomic analyses of cortical microglia revealed that 72 genes were expressed at higher levels and 27 at lower levels in males compared to females [79]. In human samples, single-nucleus RNA sequencing identified an AD-pathology-associated microglial subpopulation enriched in women [83]. In the present study, we observed similar total and activated microglial densities in both males and females across NC and AD groups. Therefore, if the MTG exhibits AD-related sex differences in microglial expression, they are not reflected in microglial densities.

Microglial cells have been shown to directly or indirectly influence cognitive processes [84]. In animal studies, the clodronate-induced elimination of hippocampal microglia impaired the performance in spatial memory tests. Notably, these impairments were reversed following microglial repopulation [85]. In contrast, numerous studies in animal models and humans suggest that microglial activation contributes to cognitive deficits in conditions such as schizophrenia, type 2 diabetes, amyotrophic lateral sclerosis, Parkinson’s disease, and AD [86–90].

Although no associations were found between total or activated microglial densities and global cognitive scores, we observed significant relationships with executive function. In individuals with NC, total microglial densities were positively associated with executive function scores across all MTG layers, with additional positive associations with activated microglial densities in layers 1 and 2. In individuals with AD, both total and activated microglial densities were negatively associated with executive function across all MTG layers. Notably, a similar postmortem study reported a negative association between microglial activation in the MTG and executive function in amyotrophic lateral sclerosis [86]. These findings suggest that in individuals with normal cognition, the homeostatic role of microglia supports MTG-related processes involved in executive function. In contrast, in AD, microglial activation might contribute to a dysregulated cellular environment, impairing these executive functions.

We found that both male and female AD individuals showed increased PAM inclusions across all ROIs. The increase in PAM expression occurred despite the lack of changes in the total or activated microglial populations. Accordingly, higher microglial densities were associated with amyloid plaques in many animal models [91–93]. Furthermore, postmortem studies in humans with AD have shown a positive correlation between microglial cell density near Aβ plaques and disease duration [94].

PAMs promote Aβ plaque compaction and insulation in a Trem2-dependent manner [95]. Deletion of Trem2 in mouse models of AD reduced initial Aβ plaque build-up but increased plaque deposition at later stages [96]. In the 5xFAD mouse model of AD, the Aβ accumulation in PAMs causes their death, leading to the release of Aβ into the extracellular space and promoting plaque growth [97]. Gene expression profiling has shown upregulation of proinflammatory genes in PAM from 5xFAD mice, suggesting this cell type contributes to chronic neuroinflammation in AD [98]. For instance, inositol polyphosphate-5-phosphatase (INPP5D) is a microglia-related genetic risk factor for late-onset AD that regulates inflammasome activity and is selectively expressed by PAMs [99–101]. In summary, evidence suggests that PAMs play a dual role, both limiting Aβ plaque spread at early AD stages and promoting detrimental neuroinflammatory processes at later stages.

To the best of our knowledge, this is the first study exploring the relationship between PAM expression and cognition in humans. There was a previous indication that PAM expression could be detrimental to cognition [102]. In the 3xTg-AD mouse model of AD, the inhibition of microglial association with Aβ improved memory-related cognitive performance [102]. Here, we corroborate these findings in humans. We observed an inverse relationship between the percentage of PAM inclusions with both CASI and MMSE scores in AD subjects. This relationship with global scores was observed exclusively in L5/6 of the MTG. Furthermore, cognitive domain-specific analysis revealed an inverse association between the PAM inclusions in L3 and L5/6 and memory scores, with no such associations observed for executive function, language, or visuospatial processing. These findings appear to be specific of PAM expression, as memory scores were not associated with any parameters of Aβ or microglial densities.

The selective targeting of PAMs has emerged as a promising therapeutic avenue for addressing AD pathophysiology. Pharmacological inhibition of INPP5D has been proposed as a strategy to shift microglia toward a beneficial phenotype in AD [100]. Additionally, the dendranib precision nanomedicine, based on hydroxyl dendrimers capable of crossing the blood-brain barrier in regions of inflammation, offers a novel approach [103], [104]. Recent studies in 5xFAD mice demonstrated that hydroxyl dendrimers are preferentially internalized by PAMs while sparing non-plaque-associated microglia [25]. Furthermore, combining hydroxyl dendrimers with an inhibitor of the colony-stimulating factor 1 receptor has shown promising results in mitigating AD plaque pathology, reducing microglial numbers, and decreasing plaque association [25]. The findings of this study suggest that L5/6 of the human MTG could be explored as a hotspot for histopathological evaluations of therapeutic interventions targeting PAM expression to enhance cognitive function in AD.

This study has limitations inherent to the use of data retrieved from autopsy-based studies available in online databases. Detailed information regarding the cause of death of the donors was not available, limiting further investigation into survivorship bias. The sample included in the present study was uneven in gender representation, consisting of 75 individuals, 39 with NC (23 female, 16 male) and 36 with AD (23 female, 13 male). We addressed this concern by stratifying our group analyses and adjusting our regression models by sex. It also should be noted that data on the evaluation of specific cognitive domains were available for only four domains, limiting the broader interpretation of the findings.

Moreover, the database provides morphometric analyses of cell and plaque densities instead of stereological estimates of absolute numbers. As stereological analysis accounts for the potential biases of the tridimensional organization of the objects, they are considered the optimal methodology for quantitative histology. In the discussion section, we addressed some discrepancies found between stereology studies and 2D-based quantifications in the context of this research. Additionally, quantifications were performed on a single MTG section per individual. Ideally, analyzing multiple sections per case would enhance the accuracy and reliability of the measurements.

Another limitation of the current approach is that Aβ deposits were not subclassified into diffuse or focal plaques. Since diffuse plaques are less likely to be associated with cognitive decline [51], their inclusion may have attenuated the strength of observed associations with cognition. Additionally, as the 6E10 antibody detects both APP and Aβ, we were unable to distinguish between intracellular and extracellular aggregates. As a result, we could not determine which associations with cognitive decline were specifically driven by extracellular Aβ pathology. Finally, microglial activation was inferred based on morphology using the HALO Microglia Module, which classifies IBA1+ cells with enlarged somata and retracted processes as morphologically activated. However, IBA1 is a pan-microglial marker and does not distinguish between functional activation states. The use of additional markers specific to microglial activation, such as CD68 or iNOS, would provide a more reliable classification of microglial immunophenotypes.

In conclusion, this study provides novel insights into histopathological alterations within the MTG in normal aging and AD. Significant AD-related increases in amyloidosis, average plaque areas, Aβ densities, and the percentage of PAM inclusions were observed across all cortical layers in both sexes. In contrast, the total and activated microglial densities of the MTG remained stable in AD. In layers 3 and 4 of the MTG, the size of Aβ plaques—rather than their densities or load—was negatively associated with MMSE scores in AD. Aβ plaque size in layer 2 was also negatively associated with language scores in AD. This suggests that plaque growth, rather than the quantity of Aβ plaques, in the MTG is a relevant factor for cognitive decline. Both CASI and MMSE scores were inversely correlated with PAM inclusions in layers 5 and 6 of the MTG. Moreover, PAM inclusions in L3 and L5/6 were negatively associated with memory scores. These relationships were unique to PAM expression, as no such associations with memory scores were observed with Aβ or microglial parameters. These findings suggest that the MTG could serve as a valuable target for emerging therapeutic interventions or diagnostic strategies.

## Supplementary Materials

The Supplementary data can be found online at: www.aginganddisease.org/EN/10.14336/AD.2025.0409.

## Data Availability

All data analyzed in this study are publicly available for download in this website: https://portal.brain-map.org/explore/seattle-alzheimers-disease/seattle-alzheimers-disease-brain-cell-atlas-download?edit&amplanguage=en. In our supplemental material we also detail which specific data from this online resource we analyzed.
