# Supplementary material for: Layer-Specific Colocalization of Microglia with Amyloid Plaques in the Middle Temporal Gyrus Predicts Cognitive Decline in Alzheimer's Disease
Source: Aging Dis. 2025 May 9;17(3):1568–89. doi: 10.14336/AD.2025.0409 (PMC13061560; doi:10.14336/AD.2025.0409)
Supplement: Supplementary file 1 — The Supplementary data can be found online at: www.aginganddisease.org/EN/10.14336/AD.2025.0409. [file AD-17-3-1568-s.pdf]

SUPPLEMENTARY DATA

Layer-Specific Colocalization of Microglia with Amyloid Plaques in the Middle Temporal Gyrus Predicts Cognitive Decline in Alzheimer's Disease.

Wellydo Kesllowd Marinho Escarião, Guilherme Henrique Viana da Silva, Hellen Suzane Clemente de Castro, Sayonara Pereira da Silva, Nelyane Nayara Martins de Santana, Ramon Hypolito Lima, Felipe Porto Fiuza

© 2025. Escarião WKM et al. Published online at http://www.aginganddisease.org/EN/10.14336/AD.2025.0409

SUPPLEMENTARY DATA

Donor ID	Cognitive Status	Cohort	Age (years)	Sex	Education (years)	APOE4	Last CASI Score	Interval from last CASI (months)	Last MMSE Score	Interval from last MMSE (months)	PMI	ADNC	CPS	Thal	Braak	CERAD
H20.33.034	Normal Cognition	ACT	85	Female	16	0	99	17.5	30	17.5	10.02	Intermediate	0.368	Thal 3	Braak IV	Absent
H21.33.025	Normal Cognition	ACT	88	Female	21	0	94	8.5	29	8.5	8.6	Intermediate	0.518	Thal 3	Braak IV	Moderate
H20.33.012	Normal Cognition	ACT	91	Female	18	0	98	21.8	29	21.8	7.72	Low	0.237	Thal 1	Braak III	Absent
H21.33.032	Normal Cognition	ADRC Clinical Core	98	Female	16	0			30	73.9	5	Low	0.38	Thal 2	Braak IV	Sparse
H21.33.037	Normal Cognition	ACT	88	Female	18	0	96	29.7	26	29.7	6.5	Low	0.18	Thal 2	Braak IV	Absent
H21.33.038	Normal Cognition	ACT	84	Female	15	0	97	21.2	27	21.2	11	Low	0.182	Thal 1	Braak III	Absent
H19.33.004	Normal Cognition	ACT	80	Female	17	0	85	3.5	25	3.5	8.13	Not AD	0.302	Thal 0	Braak IV	Absent
H20.33.002	Normal Cognition	ACT	97	Female	12	0	93	46.1	33	22.6	4.33	Not AD	0.207	Thal 0	Braak IV	Absent
H20.33.035	Normal Cognition	ACT	99	Female	16	0	92	3	24	3	4.6	Not AD	0.204	Thal 0	Braak IV	Absent
H21.33.041	Normal Cognition	ACT	98	Female	12	0	98	28.2	29	28.2	7.5	Not AD	0.304	Thal 0	Braak IV	Absent
H21.33.011	Normal Cognition	ACT	83	Female	17	0	91	18.2	25	18.2	3.6	Not AD	0.171	Thal 0	Braak IV	Absent
H21.33.035	Normal Cognition	ACT	97	Female	14	0	97	30	27	30	4.8	High	0.377	Thal 5	Braak V	Moderate
H20.33.039	Normal Cognition	ACT	96	Female	14	0	91	3.5	26	3.5	10.3	High	0.371	Thal 4	Braak V	Frequent
H20.33.005	Normal Cognition	ACT	99	Female	12	0	94	24.2	29	24.2	7.6	Intermediate	0.761	Thal 3	Braak IV	Moderate
H20.33.014	Normal Cognition	ADRC Clinical Core	82	Female	16	0					7.4	Intermediate	0.678	Thal 3	Braak IV	Sparse
H20.33.019	Normal Cognition	ACT	87	Female	15	1	98	22.5	30	22.5	11.1	Intermediate	0.728	Thal 4	Braak III	Sparse
H20.33.027	Normal Cognition	ACT	99	Female	17	0	88	6.7	23	6.7	9.23	Intermediate	0.626	Thal 3	Braak IV	Moderate
H21.33.022	Normal Cognition	ACT	82	Female	12	0	97	8.6	30	8.6	4.83	Intermediate	0.74	Thal 2	Braak VI	Moderate
H20.33.008	Normal Cognition	ACT	92	Female	18	1	92	2.7	29	2.7	7	High	0.754	Thal 4	Braak V	Moderate
H21.33.026	Normal Cognition	ADRC Clinical Core	90	Female	16	1			30	91.2	4.1	High	0.75	Thal 4	Braak V	Frequent
H21.33.033	Normal Cognition	ACT	83	Female	14	1	96	11.4	27	11.4	8.2	High	0.9	Thal 5	Braak V	Moderate
H21.33.036	Normal Cognition	ACT	93	Female	14	0	84	27.5	21	27.5	4	High	0.69	Thal 4	Braak V	Frequent
H20.33.030	Normal Cognition	ACT	86	Female	16	1	74	0.7	25	22.8	6	High	0.787	Thal 4	Braak V	Frequent
H20.33.024	Normal Cognition	ACT	90	Male	19	0	94	28.4	28	28.4	3.5	Intermediate	0.324	Thal 4	Braak V	Sparse
H21.33.030	Normal Cognition	ACT	89	Male	17	1	99	127.2	27	55.2	5.5	Intermediate	0.252	Thal 3	Braak III	Moderate
H21.33.047	Normal Cognition	ACT	90	Male	21	0	90	7.4	26	7.4	4.4	Intermediate	0.225	Thal 2	Braak V	Frequent
H20.33.043	Normal Cognition	ACT	85	Male	20	1	95	14.8	26	14.8	4.5	Intermediate	0.442	Thal 4	Braak IV	Sparse
H20.33.001	Normal Cognition	ACT	82	Male	16	0	97	18.2	28	18.2	7.7	Low	0.523	Thal 2	Braak IV	Sparse
H21.33.015	Normal Cognition	ACT	98	Male	12	0	84	14.7	24	14.7	4.1	Low	0.231	Thal 2	Braak IV	Sparse
H21.33.028	Normal Cognition	ACT	72	Male	16	0	99	22.3	29	22.3	8.3	Low	0.3	Thal 1	Braak II	Absent
H21.33.019	Normal Cognition	ACT	75	Male	15	0	97	2.4	28	2.4	10.02	Low	0.517	Thal 1	Braak 0	Sparse
H21.33.003	Normal Cognition	ACT	78	Male	16	0	95	18.6	27	18.6	10	Not AD	0.238	Thal 0	Braak 0	Absent
H21.33.004	Normal Cognition	ACT	93	Male	18	0	89	14.5	27	14.5	5	Not AD	0.262	Thal 0	Braak II	Absent
H21.33.023	Normal Cognition	ACT	102	Male	12	0	86	47.9	24	47.9	7.83	Not AD	0.297	Thal 0	Braak IV	Absent
H20.33.025	Normal Cognition	ACT	94	Male	12	0	94	50.2	22	23.8	4.5	High	0.589	Thal 4	Braak V	Moderate
H20.33.013	Normal Cognition	ACT	94	Male	14	0	93	18.6	25	18.6	4.5	Intermediate	0.847	Thal 3	Braak IV	Moderate
H21.33.014	Normal Cognition	ADRC Clinical Core	92	Male	15	0			28	68.1	9.5	Intermediate	0.754	Thal 4	Braak IV	Sparse
H20.33.032	Normal Cognition	ACT	98	Male	17	0	91	11.4	27	11.4	6.92	High	0.818	Thal 5	Braak V	Moderate
H21.33.040	Normal Cognition	ACT	83	Male	17	1	91	22	22	22	5.6	High	0.745	Thal 4	Braak V	Frequent
H20.33.011	Alzheimer's Disease	ACT	93	Female	16	1	79	7.8	21	7.8	6	High	0.811	Thal 5	Braak V	Moderate
H20.33.018	Alzheimer's Disease	ACT	81	Female	21	1	71	81	21	81	4.5	High	0.896	Thal 5	Braak VI	Frequent
H20.33.026	Alzheimer's Disease	ADRC Clinical Core	75	Female	12	1					7	High	0.907	Thal 4	Braak VI	Frequent
H20.33.028	Alzheimer's Disease	ACT	94	Female	12	0	75	12	22	12	7.4	High	0.815	Thal 4	Braak V	Moderate
H20.33.029	Alzheimer's Disease	ACT	91	Female	13	0	77	39.9	23	39.9	6.1	High	0.613	Thal 4	Braak V	Moderate
H20.33.031	Alzheimer's Disease	ACT	87	Female	12	0	79	22.5	26	22.5	7.92	High	0.778	Thal 4	Braak VI	Frequent
H20.33.037	Alzheimer's Disease	ACT	96	Female	20	0	70	27.7	18	27.7	10	High	0.891	Thal 5	Braak V	Frequent
H20.33.045	Alzheimer's Disease	ADRC Clinical Core	77	Female	18	1			6	88.8	3.3	High	0.922	Thal 5	Braak VI	Frequent
H21.33.007	Alzheimer's Disease	ACT	86	Female	21	0	86	41.3	26	41.3	5.6	High	0.723	Thal 4	Braak V	Frequent
H21.33.008	Alzheimer's Disease	ACT	91	Female	16	0	67	88.3	21	88.3	5	High	0.79	Thal 4	Braak V	Frequent
H21.33.009	Alzheimer's Disease	ADRC Clinical Core	65	Female	16	1			11	73.2	6.2	High	0.911	Thal 5	Braak VI	Frequent
H21.33.039	Alzheimer's Disease	ACT	88	Female	13	0	70	35.1	20	35.1	6.5	High	0.729	Thal 4	Braak V	Moderate
H21.33.042	Alzheimer's Disease	ACT	91	Female	15	1	80	36.9	21	36.9	8.1	High	0.85	Thal 5	Braak V	Moderate
H21.33.002	Alzheimer's Disease	ADRC Clinical Core	70	Female	18	1					3.2	High	0.857	Thal 5	Braak VI	Frequent
H21.33.010	Alzheimer's Disease	ACT	93	Female	13	0	87	55.6	27	55.6	9.6	High	0.822	Thal 5	Braak VI	Frequent
H21.33.017	Alzheimer's Disease	ACT	92	Female	16	0	78	5.3	19	5.3	3.5	High	0.68	Thal 5	Braak V	Frequent
H21.33.045	Alzheimer's Disease	ADRC Clinical Core	94	Female	12	1			17	65.3	4	High	0.924	Thal 4	Braak VI	Frequent
H20.33.038	Alzheimer's Disease	ACT	90	Female	18	0	83	39.4	23	39.4	6.2	High	0.749	Thal 4	Braak V	Frequent
H20.33.041	Alzheimer's Disease	ACT	91	Female	18	0	86	61.7	25	61.7	5.92	High	0.653	Thal 4	Braak V	Moderate
H21.33.013	Alzheimer's Disease	ACT	94	Female	21	1	80	117.6	23	117.6	7.3	High	0.89	Thal 4	Braak V	Moderate
H21.33.034	Alzheimer's Disease	ACT	90	Female	16	1	66	47.3	18	47.3	10.8	High	0.87	Thal 5	Braak VI	Frequent
H20.33.016	Alzheimer's Disease	ADRC Clinical Core	93	Female	14	0			25	83.7	7.73	Intermediate	0.617	Thal 4	Braak IV	Moderate
H21.33.044	Alzheimer's Disease	ACT	88	Female	15	0	81	8.9	21	8.9	7	Intermediate	0.845	Thal 3	Braak VI	Frequent
H20.33.004	Alzheimer's Disease	ACT	86	Male	15	1	80	55.7	25	55.7	8.83	High	0.725	Thal 5	Braak V	Frequent
H20.33.017	Alzheimer's Disease	ADRC Clinical Core	69	Male	14	0					5.5	High	0.79	Thal 4	Braak VI	Frequent
H20.33.020	Alzheimer's Disease	ADRC Clinical Core	81	Male	12	1			14	57.6	6.63	High	0.929	Thal 5	Braak VI	Frequent
H20.33.046	Alzheimer's Disease	ACT	94	Male	21	0	68	37.8	18	1.8	8	High	0.868	Thal 5	Braak VI	Frequent
H21.33.027	Alzheimer's Disease	ACT	92	Male	18	1	71	28.2	22	28.2	6.3	High	0.687	Thal 5	Braak V	Moderate
H21.33.029	Alzheimer's Disease	ACT	89	Male	18	1	88	78.5	26	78.5	11.03	High	0.91	Thal 5	Braak V	Frequent
H21.33.046	Alzheimer's Disease	ACT	97	Male	17	0	81	22.3	22	22.3	7	High	0.766	Thal 4	Braak V	Moderate
H20.33.033	Alzheimer's Disease	ADRC Clinical Core	68	Male	18	0			25	65.8	6.5	High	0.875	Thal 5	Braak VI	Frequent
H21.33.031	Alzheimer's Disease	ADRC Clinical Core	84	Male	18	1			27	86.7	8.9	High	0.835	Thal 5	Braak V	Moderate
H20.33.015	Alzheimer's Disease	ACT	88	Male	18	0	88	48	27	48	11.42	Intermediate	0.879	Thal 3	Braak V	Moderate
H21.33.005	Alzheimer's Disease	ACT	95	Male	16	0	98	86.6	30	86.6	10.2	Intermediate	0.576	Thal 3	Braak V	Moderate
H21.33.020	Alzheimer's Disease	ACT	82	Male	21	0	77	23.8	23	23.8	10.8	High	0.911	Thal 4	Braak V	Frequent
H21.33.021	Alzheimer's Disease	ACT	99	Male	14	0	86	37.3	24	37.3	6.9	Intermediate	0.705	Thal 4	Braak III	Moderate

*3 non-demented individuals were excluded for clinical notes suggesting MCI or multiple systems atrophy (H20.33.044, H21.33.006 and H20.33.036)

*6 individuals with dementia (AD probable/possible or unknown/other types of dementia) were excluded for presenting ADNC score "Low" or CPS below0.5 (H20.33.040, H21.33.012, H21.33.001, H21.33.016, H21.33.018 and H21.33.043).

Supplementary Figure 1. Detailed information on the donors included in the present study.

© 2025. Escarião WKM et al. Published online at http://www.aginganddisease.org/EN/10.14336/AD.2025.0409
